# Study of single nucleotide polymorphism of vascular endothelium factor in patients with differentiated thyroid cancer

**DOI:** 10.1186/s40842-022-00146-x

**Published:** 2022-12-15

**Authors:** Mohamad Mohsen Motawea, Maysaa El Sayed Zaki, Maha Saif, Asmaa Osama BS Osman, Aml Mohamed Nada

**Affiliations:** 1 Internal Medicine Department, Mansoura Faculty of Medicine, Mansoura University, Mansoura, Egypt; 2 Clinical Pathology Department, Mansoura Faculty of Medicine, Mansoura University, Mansoura, Egypt; 3 Oncology Department, Mansoura Faculty of Medicine, Mansoura University, Mansoura, Egypt; 4 Clinical Pathology Department, Assuit Faculty of Medicine, Assuit University, Assiut, Egypt

**Keywords:** VEGF, Thyroid cancer, Genetic polymorphism, Differentiated thyroid cancer

## Abstract

**Background:**

Genetic alterations and high levels of the vascular endothelial growth factor (VEGF) are presumptive risk factors for differentiated thyroid cancer (DTC).

**Objective:**

This work aims to study the presence of − 634G/C polymorphism of vascular endothelial growth factor (rs2010963) and its’ serum level in patients with DTC and comparing these results with those of the control subjects.

**Material and method:**

The study was a retrograde case–control study that included seventy patients with DTCin addition to seventy apparently healthy control subjects. Blood sample was taken and subjected to study of − 634G/C VEGF polymorphism (rs2010963) by real time PCR and measurement of its’ plasma level by immunoassay kit (ELISA).

**Results:**

Regarding genotyping of VEGFA − 634G/C (rs2010963) polymorphism, there was significant increase in CG and GG genotypes (28.6%, 18.6% respectively) among patients compared to control subjects (20.0%, 4.3% respectively) and significant increase in CC genotype in control subjects (75.7%) compared to patients (52.9%), *P* = 0.001. The VEGF mean ± SD level was significantly elevated in patients compared to control subjects (1215.81 ± 225.78 versus 307.16 ± 91.81, *P* = 0.006). Moreover, there was significant increase in VEGF levels in patients with CG and GG genotypes (1295.9 ± 68.74, 1533.08 ± 109.95, respectively) compared to patients with CC genotype (1061 163.25), *P *= 0.001).

**Conclusion:**

There was significant increase in GG and CG genotypes in patients with DTC compared to control subjects which may suggest a predisposing role for these genotypes in development of DTC. Moreover, there was significant increase in serum level of vascular endothelial growth factor in patients with GG and CG genotypes which may reflect the mechanism of these genotypes in development of DTC.

## Introduction

Thyroid cancer has shown a global increase in the last decades. There are many types of thyroid cancer that differ in their frequency. The most common type is papillary carcinoma that represents around 80% of thyroid cancer in patients with age below 70 years. The second type is the follicular carcinoma that represents around 13% of thyroid cancer. These two types are known to belong to the differentiated thyroid cancer DTC. Medullary thyroid carcinoma originating from parafollicular C cells accounts for 4% of thyroid cancer. The less common type is the poorly differentiated anaplastic thyroid cancers that constitutes 2% of thyroid malignancies [1, 2].

There are multiple risk factors that may predispose to thyroid cancer such as obesity, diabetes mellitus in women, low iodine intake, ionizing radiation and family history of benign or malignant thyroid disorders [3–5].

Genetic alterations are known risk factors for thyroid cancer. Among these genetic alterations are mutations that involve the coding genes for tyrosine kinase receptors, RET and NTRK1 intracellular effectors of the MAPK pathway, a GTP-binding protein RAS and a serine-threonine kinase BRAF. These mutations are common in more than 70% of papillary carcinoma which suggest that activation of this signaling pathway is essential for tumor initiation and that the modification of a pathway single effector is sufficient for cell transformation [6, 7]. Other molecular risk factors include the predisposition of single nucleotides polymorphism (SNP) to thyroid cancer [8]. Among the predisposing factors for thyroid cancer is the SNP in the genes coding vascular endothelial growth factor, fibroblast growth factor, angiopoietins-1 and − 2 and angiogenin [9, 10].

The formation of new blood vessels, angiogenesis is required for tumor growth and metastasis [11]. The angiogenesis process forms new blood vessels from the present blood vasculature [12]. Vascular endothelial growth factor (VEGF) is one of the most important mediators of angiogenesis that leads to cancer development, invasion and metastasis [13, 14].

The gene of VEGF is found on chromosome 6p21.3 and is formed of 8 exons and 7 introns. This gene is polymorphic with thirty SNPs in the 5′- and 3′- of the un-translated region and in the promoter regions [15]. These SNP may lead to alteration in the expression of the VEGF levels [16]. The increase in VEGF levels is associated with different types of malignancies such as breast, lung, kidney, ovarian, colorectal and stomach cancer [17–19].

Previous studies revealed that G allele at − 634 position increases the transcriptional activity that increases vascular endothelial factor production. The function of G allele is dose dependent being most active with GG genotype, least with CC and intermediate with GC [20]. Previous studies reported that polymorphism at − 634G/C of VEGF gene may be considered as a risk factor for thyroid cancer [21, 22]. However, there are no data from Egypt to the best of our knowledge. This work aims to study the presence of − 634G/C polymorphism of vascular endothelial growth factor (rs2010963) in DTC and estimate VEGF serum level in DTCpatients and comparing these results with control subjects.

## Material and method

This study is a retrograde case– control study that included seventy patients with DTC recruited from Mansoura University Hospital, Egypt from January 2019 till December 2020. In addition, seventy apparently healthy controls were included. The inclusion criteria included adult patients above 18 years with diagnosis of DTC with no other malignancies. The study was approved by Mansoura Faculty of Medicine ethical committee (R.22.04.1692) and a written consent was obtained from the participating subjects.

### Diagnosis of patients

Patients were subjected to full medical history, clinical examination, and imaging in the form of computed tomography scan, ultrasound examination and core biopsy for pathological examination. The staging of DTC depends upon the tumor size, spread to lymph nodes and the presence of distant metastasis according to the American Joint Committee on Cancer [23].

### Laboratory samples

Ten microliters blood samples were obtained from each participating subject under complete aseptic technique. The sample was divided into two aliquots with added heparin. One aliquot for DNA extraction and real time PCR study of − 634G/C VEGF polymorphism (rs2010963). The other aliquot was subjected to plasma separation by centrifugation for measurement of plasma level of VEGF by immunoassay kit (Quantikine®ELISA-USA R&D Systems, Inc. 614 McKinley Place NE, Minneapolis, MN 55,413).

### Immunoassay for VEGF

The kit used the quantitative sandwich enzyme immunoassay technique. The microplate wells were coated with monoclonal antibody specific for human VEGF. The samples and standards were added to the wells then incubated so the VEGF binds to the antibodies coating the wells, then undergo washing to remove the unbound substances. A second polyclonal antibody specific for human VEGF bound to the enzyme was added to the wells and incubated, then undergo second wash to remove any unbound antibody-enzyme reagent, after that a substrate solution was added to the wells. A proportional color developed in proportion to the amount of VEGF found in the samples and in the standards. Stop reagent was added after incubation. Two readings were performed using immunoassay plate reader at 570 nm and at 450 nm. The first reading was subtracted from the second reading for correction of optical imperfections in the plate. The results were expressed as pg/ml.

### Genotyping of VEGFA − 634G/C polymorphism (rs2010963)

#### DNA extraction from blood

DNA was extracted from the blood sample using QiAamp DNA extraction kit with the silica-membrane-based DNA purification from whole blood. The extraction was performed according to the manufacturer assay (Qiagen-United Kingdom). The extracted DNA was kept at -20 °C waiting for PCR.

### Real time PCR

The amplification procedures was applied to the − 634G/C polymorphism located in the promoter region of the VEGFA gene. The used primers and probes sequences were

- ***VEGFA*-**5′-GAGAGAAGTCGAGGAAGAGAGAGA-3′ and
- 5′-CCCAAAAGCAGGTCA CTCACTT-3′
- ***VEGFA*-FAM-**5′- CCTGTCCCTTTCGC-3′ and 5′- CCTGTCGCTTTCGC-3′.

The used kit is TaqMan Genotyping Assay (Life Technologies – Applied Biosystems – ABI, Foster City, CA). The real time amplification procedures were performed with total volume 5 µL containing 2 ng genomic DNA, TaqMan Genotyping Master Mix 1× (ABI), and Custom TaqMan Genotyping Assay 1× (ABI). PCR was performed in a 7500 fast real time PCR system (ABI). The amplification procedures included an initial cycle of 95 °C for 10 min, followed by 45 cycles of 95 °C for 15 s and 60 °C for 1 min [23].

### Statistical analysis

The data of the study was analyzed by SPPS 22. Qualitative data was expressed as number and percentage and compared by chi-square test. The numerical data was expressed as mean and standard deviation (SD) and compared by one-way ANOVA test; significant results were followed by Post-hoc Turkey HSD tests The P value was considered significant if *p* < 0.05.

### Sample size

Sample size was calculated by using Power Analysis and Sample Size (PASS) Software (version 15, 2017). NCSS, LLC. Kaysville, Utah, USA. Based on a previous study by Pasieka et al. [24] in which there was a significantly higher mean concentrations of VEGF in the serum of patients with DTCcompared with the control group, we hypothesized at least a medium effect size (d = 0.5). accordingly, group sample sizes of 70 cases and 70 control subjects achieve 83.58% power to reject the null hypothesis of zero effect size when the population effect size is 0.50 and the significance level (α) is 0.050 using a two-sided two-sample equal-variance t-test.

## Results

The study included 70 patients with DTC and 70 age and sex matched control subjects. The patients were 49 (70%) females and 21 (30%) males with mean age 54.57 ± 7.73 years, and the control subjects were also 49 (70%) females and 21 (30%) males with mean age 53.61 ± 7.34 years, *P* = 0.54. The clinical stages of the patients were stages I and II (42.9% for each) and stage III (14.3%). The pathological types of the DTC were mainly papillary carcinoma in 66 patients (94.3%) and follicular carcinoma in 4 patients (5.7%). Most of the patients had tumor size > 2-4 cm (50%), followed by tumor size ≤ 2 cm (30%) and tumor size > 4 cm (20%).

Regarding genotyping of VEGFA − 634G/C (rs2010963) polymorphism, we used Hardy-Weinberg equilibrium to detect gene distribution in the statistical analysis, the control group was in Hardy-Weinberg, Table 1, there was significant increase in CG and GG genotypes (28.6%, 18.6% respectively) among patients compared to control subjects (20.0%, 4.3% respectively) and significant increase in CC genotype in control subjects (75.7%) compared to DTC patients (52.9%), *P* = 0.001. The VEGF mean ± SD level was significantly elevated in patients compared to control subjects (1215.81 ± 225.78 versus 307.16 ± 91.81, *P* = 0.006). There was significant increase in G haplotype among patients (46/140) compared to control subjects (20/140), *P* = 0.001, Table 2.

**Table 1:** Hardy-Weinberg equilibrium

**Group**	**Genotype**	**Observed**	**Expected**	***x***^**2**^	***P*-value**
**Control**	C/C	53	51.4	2.353	0.125
	C/G	14	17.1		
	G/G	3	1.4		
**Case**	C/C	37	31.6	8.7	0.003
	C/G	20	30.9		
	G/G	13	7.6		
**All**	C/C	90	81.8	14.88	0.0001
	C/G	34	50.4		
	G/G	16	7.8

**Table 2:** Comparison of genotyping of VEGFA−634G/C (rs2010963) polymorphism and serum levels of VEGF between patients and control

	**Patients (*n* = 70)**	**Control (*n* = 70)**	***P***
**VEGF (mean ± SD)**	1215.81 ± 225.78	307.16 ± 91.81	0.000*
**VEGF Genotypes (N0.-%)**			
CC	37 (52.9%)	53 (75.7%)	0.006**
CG	20 (28.6%)	14 (20.0%)	
GG	13 (18.6%)	3 (4.3%)	
**C**	94/140 (67.1%)	120/140 (85.7%)	0.001**
**G**	46/140 (32.9%)	20/140 (14.3%)	

*ANOVA test *p* significant <0.05, **Chi-square test *p* significant <0.05

There was significant increase in stage II among patients with CG and GG genotype (55%, 76.9% respectively) compared to patients with CC genotype (24.3%), and significant increase in stage III among patients with CG genotype (35%) compared to patients with CC and GG genotype (29.7%, 7.7% respectively), *P* = 0.001. Moreover, there was significant increase in VEGF levels in patients with CG and GG genotypes (1295.9 ± 68.74, 1533.08 ± 109.95, respectively) compared to patients with CC genotype (1061 ± 163.25), *P* = 0.001. The difference in thyroid tumor size and their pathological classification among different genotypes were not significant (*P* = 0.73, *P* = 0.24, respectively), Table 3.

**Table 3:** Comparison of demographic, clinical data and pathological data and VEGF levels in patients according to genotyping of VEGFA −634G/C (rs2010963) polymorphism

	**Patients with CC genotype (*n* = 37)**	**Patients with CG genotype (*n* = 20)**	**Patients with GG genotype (*n* = 13)**	***P***
**Age (mean ± SD)**	55.76 ± 7.7	53.8 ± 8.1	52.5 ± 7.2	*P* = 0.39*
**Sex**				
Male (N0(%))	11 (29.7%)	5 (25%)	5 (38.5%)	*P* = 0.71**
Female (N0(%))	26 (70.3%)	15 (75%)	8 (61.5%)	
**Clinical Stages**				
I (N0(%))	26 (70.3%)	2 (10%)	2 (15.4%)	*P* = 0.0001**
II (N0(%))	9 (24.3%)	11 (55%)	10 (76.9%)	
III (N0(%))	1 (29.7%)	7 (35%)	1 (7.7%)	
**Size of the tumor (N0(%))**				
≤ 2 cm	11 (29.7%)	7 (35%)	3 (23.1%)	*P* = 0.73**
> 2-4 cm	17 (45.9%)	11 (55%)	7 (53.8%)	
> 4 cm	9 (24.3%)	2 (10%)	3 (23.1%)	
**Pathological classification (N0(%))**				
Papillary carcinoma	36 (79.3%)	19 (95%)	11 (84.6%)	*P* = 0.24**
Follicular carcinoma	1 (2.7%)	1 (15%)	2 (15.4%)	
**VEGF pg/ml (mean ± SD)**	1061.054 ± 163.25	1295.9 ± 68.74	1533.08 ± 109.95	*P* = 0.001*

*ANOVA test, *p* significant < 0.05, **Chi-square test *p* significant < 0.05

VEGF serum level was significantly elevated in clinical stages II and III (1307.7 ± 219.44,1302.3 ± 80.15 pg/ml, respectively) compared to clinical stage I (1095.1 21.53), *P* = 0.001. The difference in serum levels of VEGF were not significant regarding sex, pathological types and the size of thyroid tumors (*P* = 0.66, *P* = 0.16, *P* = 0.75 respectively), Table 4.

**Table 4:** Comparison of VEGF levels among different clinical stages, pathological types and size of differentiated thyroid cancer

	**Mean± SD**	***P***
**Sex**		
Male	1197.43± 247.76	*P* = 0.66
Female	1223.69 ±217.92	
**Clinical Stage**		
I	1095.1± 21.53	*P* = 0.001
II	1307.7 ±219.44	
III	1302.3 ±80.15	
**Pathological Types**		
Papillary	1206.46 ±225.68	*P* = 0.16
Follicular	1370.0± 187.79	
**Size of the tumor**	1241.62± 207.22	*P *= 0.75
	1195.5± 238.53	
	1227.79± 230.98	

There was a statistically significant positive correlation between VEGF level and tumor stage of medium strength, the more aggressive the tumor stage the higher the level of VEGF, Table 5.

**Table 5:** Correlation between VEGF level and clinico-laboratory data (*N*=70)

**Characteristic**	**Correlation coefficient**	***P*-value**
**Age (years)**	-0.085	0.485
**Sex**	0.054	0.659
**Pathology**	0.169	0.161
**Tumor stage**	0.465	**< 0.001**
**Tumor size**	-0.053	0.663

## Discussion

In the present study, the mean level of VEGF was significantly elevated in patients compared to control subjects (1215.81 ± 225.78pg/ml versus 307.16 ± 91.81pg/ml, *P* = 0.006). This finding is in agreement with previous studies in adults and children with papillary and follicular thyroid carcinomas [25–27].

This study revealed significant increase of VEGF serum level in clinical stage II compared to patients in clinical stage I (*P* = 0.001). Previous studies reported significant increase in VEGF in patients with increased growth, progression, invasiveness, spread, and metastasis of DTC cells [28–30]. The increased level of VEGF indicates the hematogenous metastasis capability of thyroid malignancies [31]. However, another study showed that VEGF was decreased in patients with DTC [28]. The angiogenesis had a major role in microenvironment of the DTC with its’ implication in cancer progression, spread, and metastasis. Recent study has evaluated the association of circulating angiogenic factors with the clinical outcomes of DTC, providing noninvasive, low-cost, and safe tests that can be used in screening, diagnosis, and follow-up [32].

Regarding the genotyping of VEGFA − 634G/C (rs2010963) polymorphism, there was significant increase in CG and GG genotypes (28.6%, 18.6% respectively) among patients compared to control subjects (20.0%, 4.3% respectively) and significant increase in CC genotype in control subjects (75.7%) compared to patients (52.9%), *P* = 0.001. This finding agrees with the previous study by Salajegheh et al. reporting the association of rs2010963 with a risk of papillary thyroid carcinoma [33]. Moreover, a study by Khosroshahi et al., reported that CC genotype can play a protective role in thyroid cancer [33]. However, previous report by Hsiao et al., 2007 reported that polymorphism of VEGF had no role in thyroid cancer [34]. Previous research found that vascular endothelial growth factor polymorphisms play an important role in increasing the gene expression of the VEGF [20, 35–37]. This polymorphism leads to the change in the spatial structure of VEGF-A mRNA due to the alteration in the position of the loop containing this polymorphism relative to the CUG initiation codon. The G allele leads to the inappropriate spatial structure resulting in translation inhibition, while with the C allele, the mRNA spatial structure is favorable for translation [36, 37]. The tendency for alteration in the transcription factors also affects the two transcription factors, irf1 and irf3 tendencies for binding. These transcription factors are interferon regulator factors with tumor suppression effect in addition to activation of the innate immunity apoptosis and response to DNA damage for the factor irf1 [38, 39]. IRF3 is associated with the activation of the transcription of interferon’s alpha and beta, as well as other interferon-induced genes [40, 41]. Therefore, the polymorphism in the VEGFA − 634G/C (rs2010963) may affect the immune response and leads to the development of DTC.

In the present study, there was significant increase in VEGF levels in patients with CG and GG genotypes (1295.9 ± 68.74, 1533.08 ± 109.95, respectively) compared to patients with CC genotype (1061 ± 163.25), *P* = 0.001. Previous studies revealed that G allele at the position − 634 which is the site for binding MZF1 transcription factor significantly modifies the transcriptional activity and increases the production of VEGF. The function of G allele is dose dependent being most active with GG genotype and lowest with CC and intermediate with GC [20].

There was significant increase in VEGF level in stage II among patients with CG genotype and patients with GG genotype) compared to patients with genotype CC and significant increase in stage III among patients with CG compared to patients with CC genotype and patients with GG, *P* = 0.001. Previous study, revealed a relationship between the polymorphisms of the VEGF-pathway and clinical outcome of thyroid cancer which depends upon the clinical stage of the thyroid cancer [42], though the effect of VEGF in the angiogenesis of the tumor is vital, and is associated with effects with other angiogenic factors in late stages [28].

The size of anaplastic thyroid tumors has been found to reduce the inhibition of VEGF [43]. However, in the present study, the difference in VEGF level was not significant between different sizes of the tumors in the included patients. This finding was in agreement with previous report by Haytaoglu et al. [44].

## Conclusion

The present study highlights the polymorphism of VEGFA − 634G/C (rs2010963) in patients with DTCcompared to control subjects. There was significant increase in GG and CG genotypes in patients with DTC compared to control subjects which may suggest a predisposing role for these genotypes in the development of DTC. Moreover, there was significant increase in serum level of vascular endothelial growth factor with GG and CG genotypes in DTC patients which may reflect the mechanism of these genotypes in development of DTC.

### Limitations of the study

1. It is single center study that need to be done on a large scale including all districts in Egypt. to confirm our results.
2. The few numbers of patients with follicular carcinoma and few number with stage III differentiated thyroid cancer.

## Data Availability

https://data.mendeley.com/drafts/bsxxh4v3pk
